# Disentangling community functional components in a litter-macrodetritivore model system reveals the predominance of the mass ratio hypothesis

**DOI:** 10.1002/ece3.941

**Published:** 2014-01-20

**Authors:** Karolína Bílá, Marco Moretti, Francesco Bello, André TC Dias, Gianni B Pezzatti, Arend Raoul Van Oosten, Matty P Berg

**Affiliations:** 1 Community Ecology, Swiss Federal Research Institute WSLVia Belsoggiorno 22, Bellinzona, 6500, Switzerland; 2 Department of Biodiversity Research, Divison of Ecosystems Analysis, Global Change Research Centre AS CRNa sádkách 7, České Budějovice, 37005, Czech Republic; 3 Institute of Botany, Czech Academy of SciencesDukelská 135, Třeboň, 379 82, Czech Republic; 4 Department of Ecological Science, Faculty of Earth and Life Sciences, VU University AmsterdamDe Boelelaan 1085, Amsterdam, 1081 HV, the Netherlands; 5 Departamento de Ecologia, Instituto de Biologia Roberto Alcantara Gomes, Universidade do Estado do Rio de Janeiro – UERJRio de Janeiro, RJ, Brasil

**Keywords:** Community-weighted mean trait value, functional diversity, functional metrics, Isopoda, litter decomposition, macrodetritivores, trait dissimilarity

## Abstract

Recent investigations have shown that two components of community trait composition are important for key ecosystem processes: (i) the community-weighted mean trait value (CWM), related to the mass ratio hypothesis and dominant trait values in the community, and (ii) functional diversity (FD), related to the complementarity hypothesis and the divergence of trait values. However, no experiments controlling for the inherent dependence between CWM and FD have been conducted so far. We used a novel experimental framework to disentangle the unique and shared effects of CWM and FD in a leaf litter-macrodetritivore model system. We manipulated isopod assemblages varying in species number, CWM and FD of litter consumption rate to test the relative contribution of these community parameters in the decomposition process. We showed that CWM, but also the combination of CWM and FD, is a main factor controlling litter decomposition. When we tested individual biodiversity components separately, CWM of litter consumption rate showed a significant effect on decomposition, while FD and species richness alone did not. Our study demonstrated that (i) trait composition rather than species diversity drives litter decomposition, (ii) dominant trait values in the community (CWM) play a chief role in driving ecosystem processes, corroborating the mass ratio hypothesis, and (iii) trait dissimilarity can contribute in modulating the overall biodiversity effects. Future challenge is to assess whether the generality of our finding, that is, that dominant trait values (CWM) predominate over trait dissimilarity (FD), holds for other ecosystem processes, environmental conditions and different spatial and temporal scales.

## Introduction

The increasing use of natural resources, resulting in environmental change, species loss, and subsequently ecosystem deterioration, has become a worldwide concern (Millennium Ecosystem Assessment 2005). Several attempts have been made to identify consequences of community changes on ecosystem processes underlying important ecosystem services (Beier 2008; Loring 2008; Carpenter et al. 2009). Recent studies have highlighted that the community trait composition, that is, the distribution of trait values of species within communities, plays a key role in driving ecosystem processes (Petchey et al. 2004; Heemsbergen et al. 2004; Luck et al. 2009; Mouillot et al. 2011). Up to now, however, it is still not clear which aspect of the community trait composition chiefly drives ecosystem processes (Dias et al. 2013).

Two main hypotheses have been proposed to explain the effect of species traits on ecosystem processes (de Bello et al. 2010). First, the mass ratio hypothesis (Grime 1998) states that the effect of a species on a given ecosystem process is proportional to its relative abundance in the community. This is because the participation of species on the processes of matter transformation and energy flux is proportional to their contribution to the community biomass. Therefore, ecosystem processes should correlate with the community-weighted mean trait values (CWM hereafter). This metric defines the most frequent trait value in a community and is computed as an average of the trait values of species present in a community, weighted by their relative abundances (Garnier et al. 2004; Leps et al. 2006; Ricotta and Moretti 2011). Second, it has been shown that functional diversity, indicating the variation in species trait values in the community (FD hereafter), promotes nonadditive effects (i.e., effects not predictable from the sum of single species) on the processes of matter transformation and energy flux (Heemsbergen et al. 2004). Nonadditive effects can emerge either due to antagonistic (competition or inhibition) or synergistic interactions (complementarity or facilitation), leading to more efficient utilization of resources among coexisting species (Tilman et al. 1996; Petchey et al. 2004; Heemsbergen et al. 2004; Hooper et al. 2005; Mouillot et al. 2011).

Although CWM and FD express different aspects of community trait composition, they are not mutually exclusive and both explain a significant part of the variation in distinct ecosystem processes (Schumacher and Roscher 2009; Mouillot et al. 2011; Roscher et al. 2012; Butterfield and Suding 2013; Conti and Díaz 2013). Observational and experimental studies testing the relative importance of CWM and FD have shown that it is difficult to disentangle their unique contributions (Hooper et al. 2005; Thompson et al. 2005; Díaz et al. 2007; Mokany et al. 2008; Schumacher and Roscher 2009; Lavorel et al. 2011). Most importantly, CWM and FD are mathematically dependent to each other, so that using observational data alone does not allow disentangling their relative effects. Dias et al. (2013) suggested that specifically designed manipulative experiments are necessary to tease apart the effects of CWM and FD on ecosystem processes. Simultaneously testing the unique and shared contribution of these two functional metrics will improve our understanding on how community trait composition affects ecosystem processes. This is especially important in a context of environmental change, where shifts in species composition can promote distinct changes in CWM and FD of communities. Understanding how each of these metrics affect ecosystem functioning will allow us to predict ecosystem responses to environmental changes due to community dynamics.

Here, we present the first experimental test of the relative importance of CWM and FD on ecosystem processes. We investigated the effect of macrodetritivore assemblages on leaf litter decomposition, which is a key process determining important soil ecosystem services, such as soil structure, soil fertility, and primary productivity (Swift et al. 1979; Couûteaux et al. 1995; Chapin et al. 2011). Testing for the two main hypotheses, researchers often used communities of macrodetritivore species, such as isopods (crustacean), diplopods (Myriapoda), and snails (Mollusca) (Zimmer et al. 2005; De Oliveira et al. 2010; Vos et al. 2011; Treplin et al. 2013). We used terrestrial isopods as a model organism, as they represent a dominant component within macrodetritivores in many ecosystems around the world (Anderson 1977; Lavelle and Spain 2001). Although recent macrodetritivore diversity experiments with a focus on species traits attempted to reveal functional mechanisms of species effects on decomposition, their results were ambiguous (Hättenschwiler et al. 2005). Some studies highlight the importance of nonadditive effects, where different macrodetritivore species complementarily participate in the process of litter decomposition (Zimmer et al. 2005; De Oliveira et al. 2010; Vos et al. 2011), whereas others corroborate the mass ratio hypothesis, showing dominant species control over decomposition rates (Treplin et al. 2013).

Recently, studies showed that isopod species consume litter in different ways (e.g., scraping or biting; Vilisics et al. 2012), which can lead to distinct effects on ecosystem processes (e.g., nutrient leaching, soil respiration, litter fragmentation, and litter mass loss; Heemsbergen et al. 2004). Whether such differences are strong enough to cause nonadditive effects remains unknown. Here, we manipulated isopod assemblages varying in species number, CWM, and FD of litter consumption rate to test, for the first time, their unique and shared contributions in the decomposition process. CWM and FD were calculated based on species-specific litter consumption rates, which is a key effect trait directly related to leaf litter decomposition (Heemsbergen et al. 2004; Zimmer 2006; Vilisics et al. 2012). We expected a shared contribution of CWM and FD to litter decomposition, however, without clear predictions on which one would prevail.

## Materials and Methods

### Species selection

To test the relative importance of CWM and FD in macrodetritivore consumption rate on leaf litter decomposition, we selected four rather common terrestrial isopod species in forests in northwestern Europe, that is, *Philoscia muscorum, Porcellio scaber*, *Armadillidium vulgare*, and *Oniscus asellus*, belonging to four different families. The four species differ in body size and consumption rate (Hassall and Sutton 1977; for our own measurements see Table 1) and belong to different ecomorphological strategy groups (Schmalfuss 1984). These species often co-occur in the same habitats allowing more efficient decomposition due to different feeding mechanisms (Vilisics et al. 2012) what has most probably synergistic effects as one species prepares leaf substrate fragments for a smaller one or for species occurring in a deeper soil layer. (Wouters et al. 2000; Schmalfuss 2003; Vilisics et al. 2007; Berg et al. 2008). Adult individuals of these species were collected from a grassland on the Afsluitdijk, Kornwederzand (53°04′N, 5°20′E) or the botanical garden of the VU University, Amsterdam (52°33′N, 4°86′E), in the Netherlands in October 2011. Animals were stored in a climate room at 15°C, 75% air relative humidity (RH), and 12:12 light–dark cycle, allowing them to acclimate to the experimental conditions.

**Table 1:** Average fresh body mass of isopod species at the start of the experiment and average leaf litter consumption rate (±SD) at the end of the experiment based on the monocultures of the four selected isopod species.

Species	Fresh body mass (mg)	Litter consumption rate * (mg ind^−1^ day^−1^)
*Philoscia muscorum (Pm)*	10.22 ± 1.52	0.20 ± 0.29
*Porcellio scaber (Ps)*	43.55 ± 3.22	1.66 ± 0.29
*Oniscus asellus (Oa)*	64.65 ± 14.11	0.87 ± 0.56
*Armadillidium vulgare (Av)*	67.64 ± 8.71	1.80 ± 0.56

* Consumption rates of the four species studied significantly differ from each other except cons. Rates of *Porcellio scaber* and *Armadillidium vulgare*. *T*-test results: Pm ˜ Ps (*P *<* *0.000), Pm ˜ Oa (*P *=* *0.038), Pm ˜ Av (*P *=* *0.001), Ps ˜ Oa (*P *=* *0.017), Ps ˜ Av (*P *=* *0.622), Oa ˜ Av (*P *=* *0.023).

As a food source for the isopods, we selected *Fraxinus excelsior* leaf litter, which is a common tree species in northwestern Europe, eaten by all isopod species involved in this study. Freshly fallen leaves without any visible signs of herbivory, damage, or degradation were collected daily under trees next to the botanical garden of the VU University Amsterdam in October 2011. Leaves were air-dried at room temperature (±20°C) for 2 weeks, subsequently dried in a stove at 45°C for 3 days, and stored in a dark aerated room for a few days until the start of the experiment.

### Experimental design

The interdependence between CWM and FD poses a challenge on disentangling their relative importance for ecosystem processes. CWM and FD generally show a hump-shaped relationship so that assemblages with more extreme CWM values can have only low FD values. Dias et al. (2013) show that this problem applies to several of the most used indices of functional diversity. We used the framework of Dias et al. (2013) to select specific combinations of species for our experiment that would allow disentangling the interdependence between CWM and FD. Based on prior knowledge on leaf litter consumption rates of the four isopod species, we randomly selected orthogonal combinations of CWM and FD from simulated assemblages. Using the R function “funziona” (Dias et al. 2013), we calculated CWM and FD for simulated isopod assemblages (>5000 simulated assemblages) varying in species composition, species richness, and within biomass boundaries (assemblage biomass was not allowed to exceed 0.65 g or to be lower than 0.45 g) Next, we selected the four most distinct CWM–FD combinations: both high CWM and FD (HH), both low CWM and FD (LL), high CWM and low FD (HL), and low CWM and high FD (LH) litter consumption values. Within each CWM–FD combination, we randomly selected four different assemblages of species, but not allowing assemblages with the same or very similar species compositions. The selection procedure resulted in 16 unique assemblages, each of which was replicated ten times (Table 2). Additionally, monocultures of each isopod species were included to quantify the species-specific consumption rates, and animal-free microcosms were included to quantify microbial decomposition.

**Table 2:** Species composition of isopod assemblages: four monocultures and 16 unique assemblages. The total biomass in each assemblage was not allowed to exceed 0.65 g or to be lower than 0.45 g, which was accomplished by varying the number of individuals per species (see Method section for details). All assemblages were replicated 10 times.

Micro-cosmos Nr.	Treatment	Number of individuals per species				Total biomass (g) per treatment
		*Philoscia muscorum*	*Porcellio scaber*	*Oniscus asellus*	*Armadillidium vulgare*	
1	Monoculture	10				0.102
2	Monoculture		10			0.436
3	Monoculture			10		0.647
4	Monoculture				10	0.676
5	LL	0	0	2	6	0.572
6	LL	0	6	2	3	0.615
7	LL	5	0	2	6	0.609
8	LL	4	2	2	5	0.569
9	HL	0	0	7	2	0.499
10	HL	2	0	7	2	0.508
11	HL	8	0	7	0	0.479
12	HL	2	4	7	0	0.562
13	LH	7	0	3	5	0.570
14	LH	5	7	3	0	0.581
15	LH	6	3	3	3	0.648
16	LH	4	7	3	0	0.525
17	HH	3	0	6	3	0.540
18	HH	5	4	6	0	0.619
19	HH	3	2	6	2	0.648
20	HH	5	0	6	2	0.543

HH – high CWM and FD, LL – low CWM and FD, HL – high CWM and low FD, LH – low CWM and high FD.

Assemblages were added to PVC microcosms (12 cm diameter, 8 cm height), closed at the top with a Perspex lid with a central hole (7.5 cm diameter) with a gauze (0.25 mm mesh size) allowing air exchange, and at the bottom closed with a gauze (0.1 mm mesh size), topped with 110 mL of fine sand allowing water drainage. The sand was washed with lukewarm tap water to remove silt and dried in a stove at 100°C for 2 days. Three days before the introduction of the isopods, we added 7 g (±0.25 g) of dry *Fraxinus excelsior* leaf litter and a microbial wash to allow colonization of the leaf litter with microbes, as it has been shown that isopods consume preferably litter colonized by microbes as compared to freshly fallen leaves (Zimmer 2006). Microcosms were sprayed with 4 mL microbial wash using an automated plant sprayer followed by 2 mL tap water. For the microbial wash, 500 mL partly degraded litter was collected in a mixed deciduous forest in the vicinity of the VU University Amsterdam, placed in a bottle (2 L) filled with 1 L tap water, shaken for 1 day (150 rpm, Edmund Bühler, shaker type SM 25) and left overnight. The microbial wash was sieved the next day over subsequently 0.18-, 0.125-, and 0.063-mm metal sieves to exclude sand and course debris.

Microcosms were kept in a climate room at 15°C, 75% RH and a 12:12 h light–dark cycle, visually controlled for water loss, watered, and randomized over the climate chamber twice a week. Water lost due to evaporation was added to the microcosm, partly via the tray and taken up via the bottom of the microcosm and partly sprayed through the gauze of the Perspex lid, without disturbing the system. The experiment ran for 8 weeks until approximately 40% litter mass loss was reached in the microcosms with the fastest decomposition, ensuring that sufficient resources were available for the isopods during the entire experiment.

At the end of the experiment, we collected all isopods, separated them by species, and state (alive or dead). Mortality mainly ranged from 0 to 10%, only eight microcosms exhibited mortality higher than 50%. All isopods were frozen at −18°C and stored till processing. Isopods were freeze-dried at −40°C for 48 h (Lyph-Lock 6, Labconco) and weighted on a Mettler Toledo microbalance (till the nearest 0.01 mg) to determine their dry mass. The remaining leaf litter in the microcosms was dried in a stove at 45°C for 2 days. The difference between the start and final dry weight was used as a measure of litter mass loss and expressed as % mass loss.

### Indices and data analyses

We computed CWM and FD of litter consumption for our isopod assemblages after correction for dead animals and microbial decomposition. CWM of litter consumption was calculated as the summed species-specific consumption (based on the monoculture values) weighted by the relative abundance of the species in the assemblage (Garnier et al. 2004). As functional diversity can be expressed by different metrics, we computed three of the most used indices, that is, functional divergence (FDiv), functional richness (FRic), and functional evenness (FEve) (Villéger et al. 2008). We mostly considered functional divergence (FDiv) in our computations as an aspect of FD reflecting the abundance distribution of species trait values in an assemblage. In our case, it captures the degree of divergence in consumption rate in an assemblage (Villéger et al. 2008). FDiv was calculated as Rao's quadratic entropy based on pairwise species dissimilarity using Gower's distance (Ricotta and Moretti 2011). We separately tested effects of two additional FD indices, functional richness (FRic), and functional evenness (FEve), to assess all possible aspects of the variation in trait values within assemblages (Villéger et al. 2008; Mouillot et al. 2011). These indices did not provide different results and were therefore included only in the Supporting Information (Fig. S1).

We excluded microcosms exhibiting isopod mortality higher than 50% (8 from 184 microcosms in total) from the analyses. The mortality rate was estimated as the proportion of dead animals at the end of the experiment to the number of individuals added at the start. For each monoculture and assemblage, litter consumption by the isopods was expressed as the total mass of leaf litter consumed per initial number of individuals. From the animal-free microcosms, we calculated the average microbial decomposition, that is, leaf litter mass respired by microbes (30.38 ± 5.08 mg d^−1^), and subtracted this value from the overall litter consumption to obtain the contribution of isopods to decomposition.

To quantify the relative importance of isopod CWM and FD leaf litter consumption on litter mass loss, we used linear mixed-effects models (Verbeke and Molenberghs 2000; McCulloch and Searle 2001). The selected species assemblages were not independent, and thus, the 16 unique assemblages were set as random effects in the model. Using the R package *glmulti* (Calcagno and de Mazancourt 2010), all possible combinations of the explanatory variables (CWM, FDiv and SR) were evaluated. All computed models were ranked on the base of the AICc coefficient (Akaike information criterion with a second order correction for small sample size), calculated using the maximum likelihood estimation (ML). The most informative models showing the lowest AICc values were analyzed more in detail. Finally, we fitted with a simple linear regression the relationships of individual explanatory variables (CWM, FDiv and SR) and leaf litter mass loss, considering only the average values of the different 16 species combinations to ensure data independence. Data analyses were performed using R version 2.15.0 (R Development Core Team 2012).

## Results

First, we attempted to elucidate functional mechanisms driving litter decomposition and tested all community components, that is, CWM, FDiv, and SR, simultaneously. The best linear mixed-effects model selected CWM of isopod litter consumption rate as the best explanatory variable for the leaf litter mass loss, showing the lowest AICc value and highest weight (Table 3). The mixed-effect models identified combinations of both CWM and FDiv (second best model), and CWM and SR (third best model) as important determinants for litter decomposition. Nonetheless, estimated *t*-values (Table 4) of the fixed effects showed best results for CWM (same as in models 1–3).

**Table 3:** Best linear mixed-effects models of variability of leaf litter mass loss within assemblages with different CWM-FD combinations, that is, both high CWM and FD (HH), both low CWM and FD (LL), high CWM and low FD (HL), and low CWM and high FD (LH) values (with ML estimation). Model ranking is based on AICc value with fixed effects of community-weighted mean (CWM), functional diversity calculated as functional divergence (FDiv) and species richness (SR). Model 5 is the null model with intercept only.

Model	log_Lik_	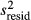	*s*_resid_	AICc	Weights
1. Massloss˜1 + CWM	−433.16	14.297	3.781	874.58	0.362
2. Massloss˜1 + CWM + FDiv	−425.36	14.302	3.782	875.50	0.228
3. Massloss˜1 + CWM + SR	−427.34	14.308	3.783	875.86	0.190
4. Massloss˜1 + CWM + FDiv + SR	−424.25	14.306	3.782	877.62	0.079
5. Massloss˜1	−435.82	14.306	3.782	877.80	0.072

**Table 4:** Estimates of the fixed effects and intercept for model 4, including all explanatory variables tested, showing the magnitude of their effects on litter mass loss.

	Estimate	SE	*t*-value
Intercept	5.012	3.555	1.410
CWM	98.428	41.716	2.360
FDiv	4.303	7.567	0.569
SR	0.209	1.216	0.172

**Figure 1:**
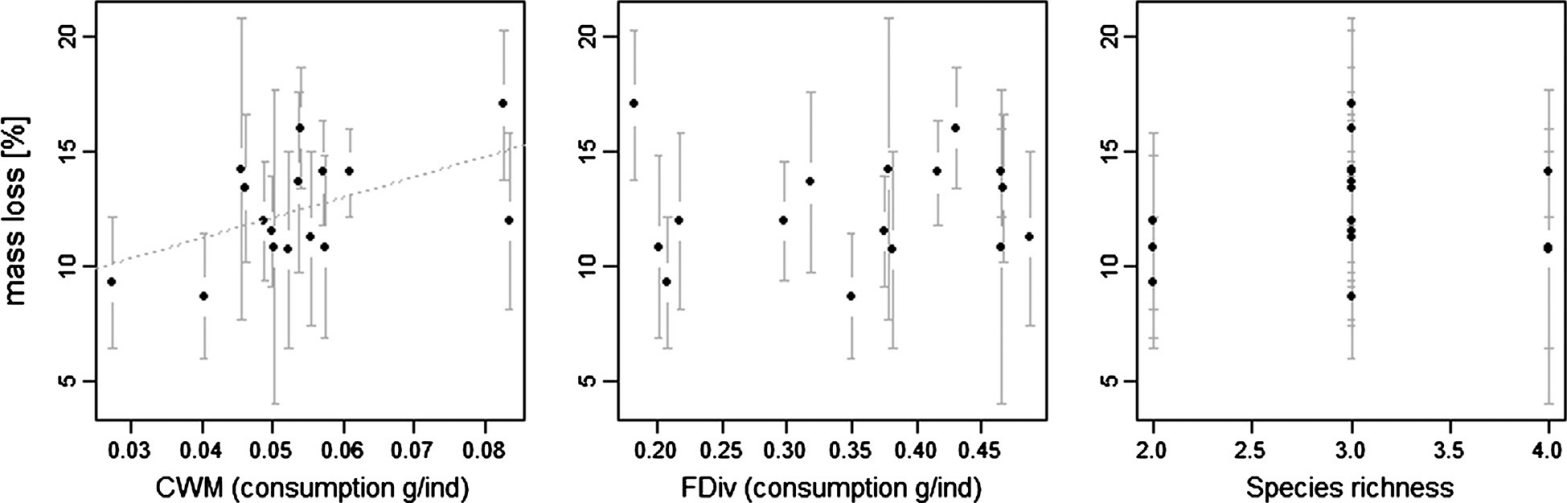
Relationships between all explanatory variables tested individually, that is, isopod community-weighted mean (CWM) consumption rate (consumption g ind^−1^), functional diversity calculated as functional divergence (FDiv) of consumption rates by isopods (consumption g ind^−1^) and species richness and leaf litter mass loss (%). Only CWM showed a significant positive effect on leaf litter mass loss (linear regression, *P *=* *0.032), whereas FDiv and species richness were not significant (linear regression, *P *>* *0.1).

Second, we tested the above-mentioned community components separately to describe their effects on litter decomposition. Relationships between individual explanatory variables, that is, CWM, FDiv, and SR, and leaf litter mass loss were tested by linear regression and showed a significant positive effect for CWM (*P *=* *0.032), whereas FDiv and SR did not significantly affect litter mass loss (Fig. 1).

## Discussion

Our results indicate that leaf litter decomposition was mainly affected by CWM, corroborating the mass ratio hypothesis (Grime 1998). This means that species with the most frequent trait values in the community, that is,the dominant species, are the main driver of litter decomposition.

Functional diversity, calculated using three different indices capturing different aspects of trait variability, showed no significant effect on litter decomposition when FD was considered alone. However, FD did complement CWM in explaining the decomposition process when CWM and FD were included in the same model. The marginal importance of FD in combination with CWM indicates that FD complements CWM, but that nonadditive effects related to antagonistic or synergistic interactions among isopod species were weak in our model system. This suggests that if species have similar abundances in the community, hence have equal weight on processes according to the mass ratio hypothesis, FD might further modulate ecosystem processes rates.

Moreover, isopod species richness did not affect litter decomposition, which is in support to recent studies (Heemsbergen et al. 2004; Mouillot et al. 2011). Although, the variation in species richness in our experiment was rather small, effects of species diversity on ecosystem processes are typically stronger on the lower range of species richness (Heemsbergen et al. 2004; Bady et al. 2005; Hector and Bagchi 2007; Gamfeldt et al. 2008). Therefore, we conclude that the functional trait composition of a macrodetritivore community plays a stronger role on determining decomposition as compared to taxonomic diversity.

As far as we know, our study is the first to decouple experimentally the relative importance of CWM and FD on driving ecosystem processes what helps us to better understand their unique versus shared contribution in the given process. The fact that CWM dominates over FD in this study confirms several existing studies (see Introduction and de Bello et al. 2010 for a review) although it contradicts, to some extent, some studies showing that FD of macrodetritivores can have a strong effect on litter mass loss (Mermillod-Blondin et al. 2002; Heemsbergen et al. 2004). It should be noted, however, that none of these studies controlled for the potential effects of CWM and its linkages with FD. In the study by Heemsbergen et al. (2004), a positive correlation was observed between dissimilarity in consumption rate in macrodetritivore assemblages and litter mass loss. However, in that study, all species in the assemblages had equal abundances or biomass, which maximizes potential FD effects on litter decomposition. Contrary to our primary expectations, we did not find nonadditive effects of isopod communities on litter decomposition, even though our selection was based on their morphological and physiological dissimilarities. We cannot exclude that an increase in the range in trait values of our selected species was too small to infer nonadditive effects, and including even more dissimilar species (i.e., small soil-dwelling species) might give different results. It also has to be mentioned that most of the published studies explore the variation between taxonomic groups of macrodetritivores; however, our study focuses on the trait variation within one taxonomic group (isopods) only. For example, Vos et al. (2011) found nonadditive effects important, when litter decomposition rate increased with the number of macrodetritivores taxonomic groups (earthworms and woodlice). These nonadditive effects were explained by distinct food preferences, with species belonging to separate groups feeding either on slow or fast decomposing litter. Synergistic effects of woodlice and earthworms were also found by Zimmer et al. (2005) who pointed out the importance of the quality and diversity of the food sources for nonadditive effects, and species-specific characteristics of the macrodetritivores. A positive complementarity effect on litter decomposition was also observed by De Oliveira et al. (2010) when offering leaf litter (single or mixed species) to a gastropod and a diplopod, kept separately or together. Another study, on the contrary, corroborated our results by testing land snails together with litter consuming crabs where the species mixture did not increase decomposition rates and decomposition was rather controlled by the dominant species (Treplin et al. 2013). Similarly, Heemsbergen et al. (2004) showed that species identity was more important for decomposition processes than both species number and taxon diversity. These examples show the necessity to consider both community components, CWM and FD, to correctly enclose different aspects of functional diversity and their unique and shared effects on distinct ecosystem processes.

Our results suggest that accounting for the effect of CWM using experimental manipulation to disentangle it from FD might give a novel insight on the mechanisms driving ecosystem functions. While in our study the effect of CWM was stronger than FD, the relative importance of CWM and FD may highly depend on the ecosystem process or services of interest (Mouillot et al. 2011). While CWM has been shown to determine litter decomposition and plant primary productivity (Lavorel and Garnier 2002; Bokhorst et al. 2010; de Bello et al. 2010; Makkonen et al. 2011), FD may be of major significance in pollinator communities, where it can significantly enhance pollination services (Albrecht et al. 2012). Additionally, some studies have shown that both CWM and FD significantly impact ecosystem processes. For instance, Schumacher and Roscher (2009) reported that both CWM and FD of multiple plant traits increase the amount of explained variance of aboveground vegetation biomass. Furthermore, Ibanez et al. (2013) and Moretti et al. (2013) have shown that grasshopper species both respond to the dominant plant traits (CWM), satisfying the bulk nutritional needs and biomechanical constraints, and plant trait dissimilarity (FD), satisfying species-specific nutritional needs. The combined importance of CWM and FD is particularly essential in communities comprised of specialized species, whereas generalists, such as our four isopod species, exhibit a different example of resource–host relationship so that their feeding efficiency, that is, CWM consumption rate, is the driver of litter decomposition rather than nonadditive (synergistic or antagonistic) effects. However, even in this case, CWM together with FD contributes to explaining the ecosystem process studied. The same pattern was found for multiple ecosystem processes including litter decomposition and plant productivity by Mouillot et al. (2011), who also suggested considering both CWM and FD to comprehensively understand the role of functional components of ecosystem processes. Future challenges are to reveal under which circumstances CWM or FD drive single ecosystem processes, and if the relative importance of the two functional components are always the same when multiple ecosystem processes are addressed or when multiple effect traits or trait syndromes are considered.

## Conclusions

Our experiment disentangled the unique contributions of CWM and FD in effect traits on a key ecosystem process and proved that CWM of litter consumption is the most important functional community component in the litter-macrodetritivore model system. We recommend applying the used methodological approach to future biodiversity–ecosystem functioning experiments, enabling to detect causal relationships between these two community components. Future research should be directed at assessing whether the generality of our finding that CWM predominates over FD holds under specific conditions, at different spatial and temporal scales, for specific ecosystem processes or simultaneously for multiple processes. Detecting general rules of community functioning might elucidate future scenarios of biodiversity threats due to global changes as increasing climatic extremes or biological invasions and thus help us to prevent further ecosystems' impoverishment.

## Supporting Information

Fig S1 Relationships of functional richness (FRic) and functional eveness (FEve) with leaf litter mass loss (mass loss %).
