# Supplementary material for: Disentangling community functional components in a litter-macrodetritivore model system reveals the predominance of the mass ratio hypothesis
Source: Ecol Evol. 2014 Jan 20;4(4):408–16. doi: 10.1002/ece3.941 (PMC3936387; doi:10.1002/ece3.941)
Supplement: Fig S1 — Relationships of functional richness (FRic) and functional eveness (FEve) with leaf litter mass loss (mass loss %). [file ece30004-0408-sd1.docx]

**Supporting Information**


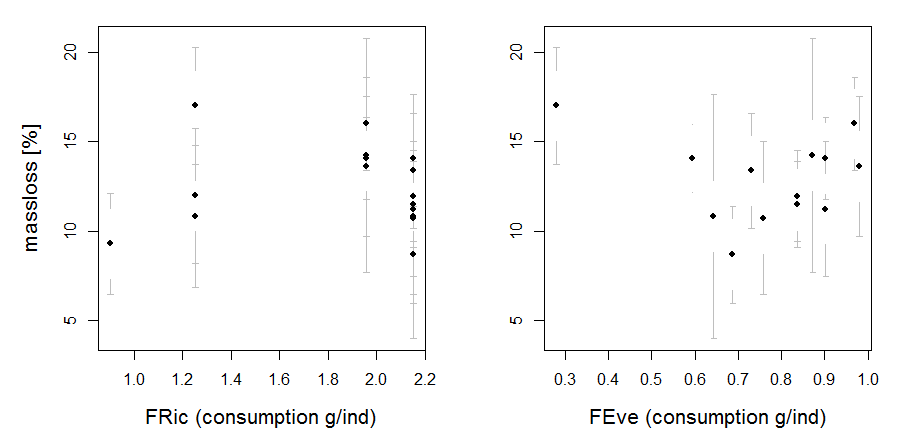


Fig. S1. Relationships of functional richness (FRic) and functional eveness (FEve) with leaf litter mass loss (mass loss %). The two functional indices related to variation in the trait consumption rate (g ind^-1^) by isopods. FRic considers the range of trait values present in the assemblage, thus the difference between the maximum and minimum consumption rate. FEve describes the evenness of abundance distribution across consumption rate of the single species. Both indices showed non-significant relationships with litter mass loss (*p* > 0.1).
